# Evolution of microRNA in primates

**DOI:** 10.1371/journal.pone.0176596

**Published:** 2017-06-22

**Authors:** Jey C. McCreight, Sean E. Schneider, Damien B. Wilburn, Willie J. Swanson

**Affiliations:** 1 Department of Genome Sciences, University of Washington, Seattle, Washington, United States of America; 2 Fred Hutchinson Cancer Research Center, Seattle, Washington, United States of America; Kunming University of Science and Technology, CHINA

## Abstract

MicroRNA play an important role in post-transcriptional regulation of most transcripts in the human genome, but their evolution across the primate lineage is largely uncharacterized. A particular miRNA can have one to thousands of messenger RNA targets, establishing the potential for a small change in sequence or overall miRNA structure to have profound phenotypic effects. However, the majority of non-human primate miRNA is predicted solely by homology to the human genome and lacks experimental validation. In the present study, we sequenced thirteen species representing a wide range of the primate phylogeny. Hundreds of miRNA were validated, and the number of species with experimentally validated miRNA was tripled. These species include a sister taxon to humans (bonobo) and basal primates (aye-aye, mouse lemur, galago). Consistent with previous studies, we found the seed region and mature miRNA to be highly conserved across primates, with overall structural conservation of the pre-miRNA hairpin. However, there were a number of interesting exceptions, including a seed shift due to structural changes in miR-501. We also identified an increase in the number of miR-320 paralogs throughout primate evolution. Many of these non-conserved miRNA appear to regulate neuronal processes, illustrating the importance of investigating miRNA to learn more about human evolution.

## Introduction

Comparative genomics is an indispensable tool for studying the evolutionary history of any organism. Humans are no exception: people are perpetually fascinated with the molecular phenotypes that differentiate us from other primates. Studies comparing protein coding sequence data has uncovered rapid evolution between primates in many key areas, including immunity, sensory perception, reproduction, and keratinization [1, 2, 3]. However, results from genomic scale analyses continue to reinforce that much, if not most, phenotypic differences between species result from changes in gene expression and not amino acid divergence [3, 4, 5, 6, 7]. While there are many layers of gene regulation that exist between DNA sequence data and expressed proteins, emphasis is often placed on the mechanisms that regulate transcription. There has been much work characterizing the coevolution of transcription factors and their DNA binding elements [8]. However, less is known concerning the evolution of post-transcriptional regulatory elements. In addition to RNA binding proteins, microRNAs (miRNA) are an important class of post-transcriptional trans-acting factors that regulate mRNA stability and rates of translation [9]. Despite their critical importance in seemingly every biological process (cell proliferation, differentiation, metabolism, apoptosis) [10], the role of miRNA in primate evolution has yet to be thoroughly examined. In this manuscript we provide an in depth characterization of miRNA identification and evolution across multiple primate lineages.

### MicroRNA biochemistry

MiRNAs are short, noncoding, single-stranded RNAs important for post-transcriptional regulation in eukaryotes. MiRNAs are a relatively new addition to our understanding of genetics: the first miRNA was discovered in *Caenorhabditis elegans* in 1993, but the widespread effects of miRNAs were not fully recognized until the early 2000s [11]. Since then, discoveries in the miRNA field have expanded our understanding of genetics, illustrating the complexity of regulatory networks and the interplay between sequence and structure. Phylogenetic studies have shown that miRNAs have been present throughout the evolution of metazoans and that increased number and expression of miRNAs are positively associated with structural and organismal complexity [6, 11]. Non-conserved miRNAs can be an indicator of adaptation in the genome of an organism, leading to novel phenotypes and a number of diseases, including heart disease, schizophrenia, and numerous types of cancers [6, 10, 12].

**Fig 1:**
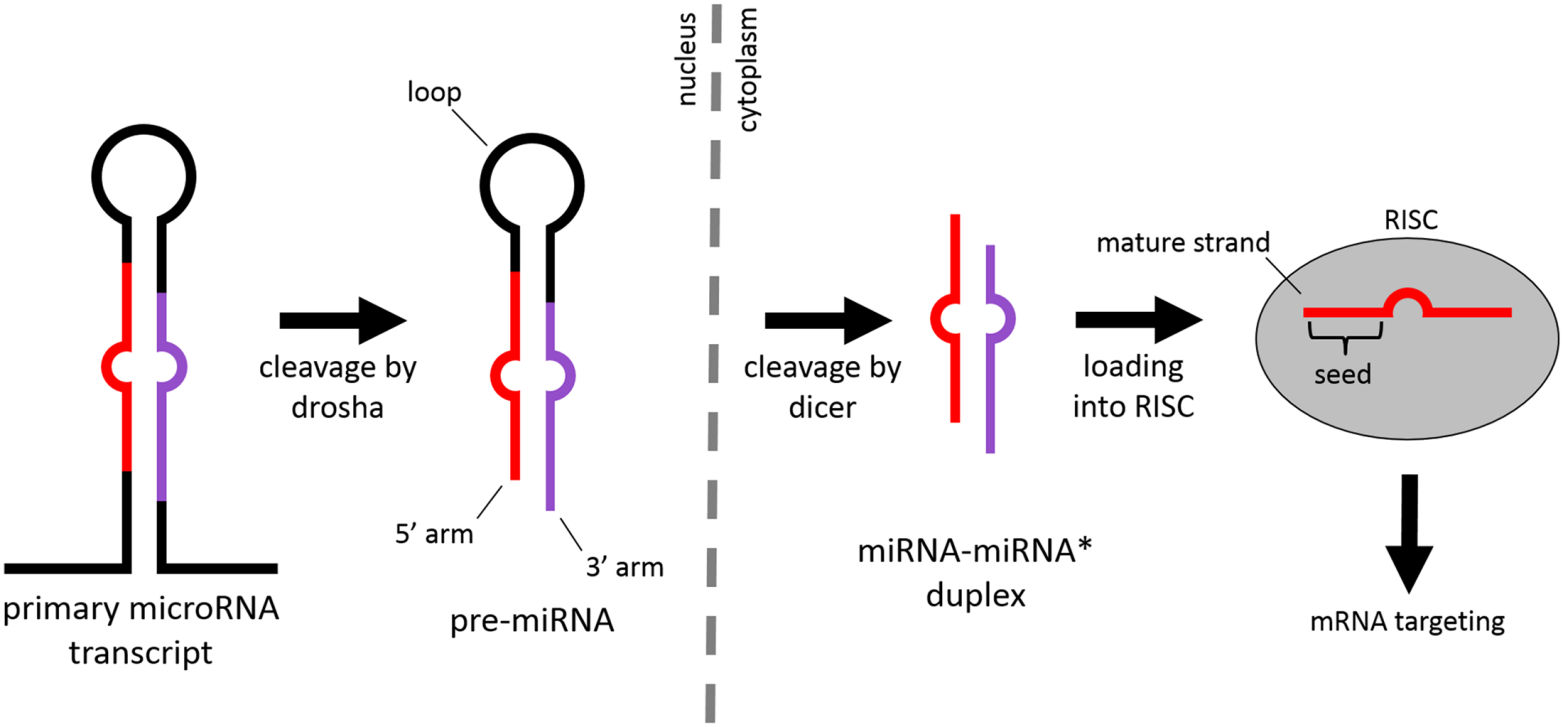
miRNA biogenesis. miRNA genes are transcribed from the genome, resulting in a primary miRNA transcript. Regions of the primary miRNA form a hairpin structure that is recognized by the endonuclease drosha, which cleaves the double-stranded stem region of the hairpin to create a pre-miRNA of ~83 nt in length. The pre-miRNA is exported to the cytoplasm where it is further processed by dicer, which cleaves off the loop region of the hairpin. This results in an approximately 22 to 23 nt double-stranded RNA called the miRNA-miRNA* duplex. The mature miRNA strand is loaded into the RNA-induced silencing complex (RISC), where its 8 nt seed region complementarily base pairs with messenger RNA targets, leading to their downregulation.

The intricate process of miRNA biogenesis (Fig 1) plays a crucial role in generating diverse phenotypes in organisms, as an alteration to any step may have profound downstream effects. MiRNA genes are transcribed from the genome, resulting in a primary miRNA transcript that may include a single miRNA or a cluster of miRNAs [11]. Regions of a primary miRNA form hairpin structures that are recognized by the endonuclease drosha, which cleaves the double-stranded stem region of the hairpin to produce an approximately 83 nucleotide (nt) precursor miRNA (pre-miRNA) [13]. After being exported to the cytoplasm, pre-miRNA are further processed by a second endonuclease, dicer, which cleaves off the loop region of the hairpin to produce an approximately 22 nt double stranded RNA duplex that contains the mature miRNA and its complement (termed the star strand, or miRNA*). The strand with the less thermodynamically stable 5’ end becomes the mature sequence and is loaded into the RNA-induced silencing complex (RISC), while the star sequence is degraded. Occasionally a pre-miRNA has both of its miRNA and miRNA* strands lead to mature sequences. The mature miRNA base-pairs with complementary sequence within the 3’ untranslated region (UTR) of messenger RNA (mRNA). This process guides RISC to specific transcripts, resulting in down-regulation of the targets through degradation of the transcripts or inhibition of translation [14]. While there are varying degrees of complementarity between a miRNA and its mRNA target, binding is most highly dependent on positions 1 through 8 of the 5’ end of the mature miRNA, known as the seed region [11]. While 75% of downregulated mRNA have canonical seed sites in their 3’ UTR, the seed region is not always sufficient for causing downregulation [15]. The 3’ end of the mature miRNA can also have an effect: positions 13–16 are highly conserved, and their proper complementary base pairing to a mRNA target is associated with downregulation [15].

About 60% of all human transcripts contain known or predicted miRNA target recognition sites [16]. A single type of miRNA can have one to thousands of targets, which establishes the potential for small changes in miRNA sequence to have profound phenotypic effects: each miRNA may result in varying degrees of phenotypic plasticity for different cell types, which have different target mRNAs to act upon. Additionally, many miRNA have multiple paralogs throughout the genome. Gene duplication followed by mutation in one copy is a common avenue for the evolution of novel functions: by maintaining more than one gene copy for a given miRNA, purifying selection to preserve function is often relaxed for one of the paralogs, allowing for mutational acquisition, differentiation, neofunctionalization, and subfunctionalization [17]. Most miRNA are highly conserved across species and show higher rates of purifying selection than protein-coding regions of the genome, suggesting that variation found in miRNA sequence may play a vital role in the evolution of metazoans [18, 19, 20, 21].

### MiRNA structure

While many studies focus on changes in miRNA expression or variants in the seed region, changes in pre-miRNA secondary structure can also dramatically affect downstream function through several different mechanisms. In general, variants in the stems of pre-miRNAs that decrease overall structural stability of the hairpin reduce the production of mature miRNA [22]. If the pre-miRNA has a secondary structure that is very divergent from the standard hairpin, the ability of drosha to recognize and process the pre-miRNA may be reduced or completely eliminated. Small changes in sequence may have drastic effects: a variant in the mature sequence of miRNA-125a blocks the processing of primary miRNA to pre-miRNA, resulting in complete loss of function [23]. However, sequence divergence does not always imply structural divergence, as compensatory mutations often help conserve a pre-miRNA’s hairpin structure.

A mutation in the primary miRNA sequence could also result in a different but stable hairpin structure. This could alter the location of drosha cleavage sites during pre-miRNA biogenesis [24], and in turn shift the cleavage sites of dicer, resulting in a different mature miRNA and thus different seed region. Sun et al. identified such a variant with an altered cleavage site and seed region shift [25]. Previous studies have only investigated a limited number of pre-miRNA variants, and the effects of most are still unknown.

### Primate miRNA diversity

**Fig 2:**
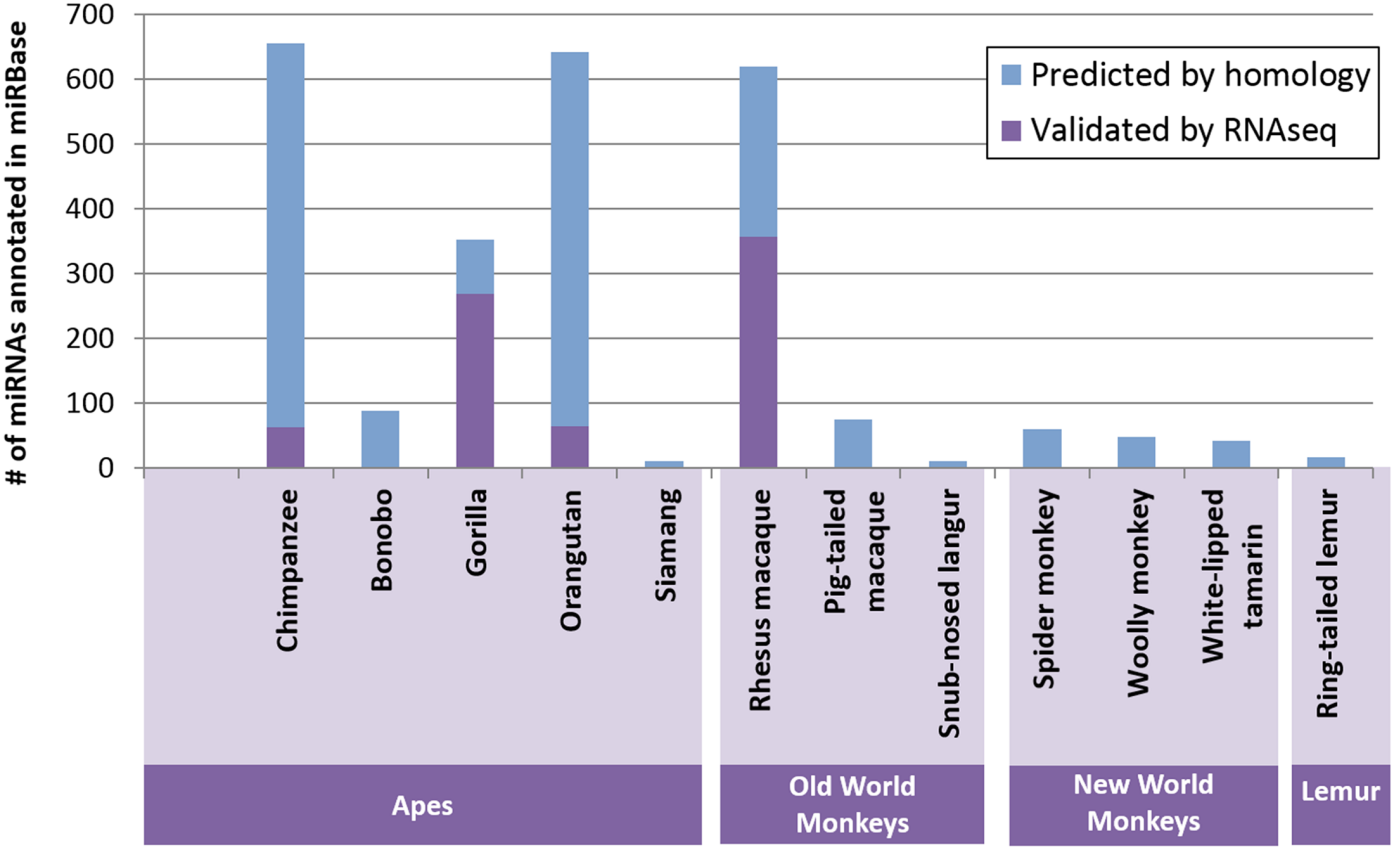
Non-human primate miRNA is poorly characterized. Only 12 of the ~300 known primate species have any entries in miRBase (release 21). The majority of these miRNA are predicted based only on homology (shown in blue); only four species (chimpanzee, gorilla, orangutan, and rhesus macaque) have sequences that are experimentally validated through RNAseq or other expression analysis (shown in purple). The number of characterized human pre-miRNAs (n = 1881) is still more than twice as large as that of chimpanzee (n = 655).

A major roadblock to studying miRNA across primates is the lack of experimentally verified miRNA in non-human primates. Only 12 of the ~300 known primate species [26] have any entries in miRBase (Fig 2, S1 Table) [27]. The number of characterized human pre-miRNAs (n = 1881) is still more than twice as large as that of chimpanzee (n = 655), the most well studied non-human primate. The majority of these miRNA are predicted based only on homology to the human genome; only four species (chimpanzee, gorilla, orangutan, and rhesus macaque) have sequences that are experimentally validated through RNAseq or other expression analysis. While homology is a useful tool for identifying orthologs, it may result in an overrepresentation of conserved sequences with respect to humans, missing sequence diversity in more distantly related primate species. Homology alone cannot identify if a sequence is actually expressed or forms a stable hairpin that successfully completes processing by drosha and dicer. Even in cases where a mature miRNA is produced, one cannot determine the exact boundaries of the mature sequence (and thus seed region) without expression data [28]. The current best mature predictive software (Mature Bayes) only comes within 1 nt of the true mature sequence 49% of the time, which can result in an incorrect seed region and thus target repertoire [29].

Despite this lack of validated miRNAs, some research has compared differences in miRNA across primates. Berezikov et al. used high throughput sequencing technology to discover miRNAs in the brains of human fetuses and chimpanzee adults [30]. While they discovered hundreds of miRNAs specific to primates with dozens not conserved between humans and chimpanzees, they did not perform any functional validation of the targets of these miRNAs. Hu et al. recently discovered several miRNA that were differentially expressed in human and chimpanzee brains, and that this differential expression resulted in downregulation of several neuronal genes [31]. Zhang et al. discovered an X-linked miRNA cluster that was rapidly evolving in primates, and these miRNA had increased expression during male sexual maturation [32].

However, techniques investigating only the expression level of miRNA would miss any phenotypic differences caused by changes in miRNA target specificity. Target specificity could change due to sequence differences in the seed region, or sequence differences that change the secondary structure of pre-miRNA and thus alter its downstream processing. Non-conserved miRNA provide insights into primate evolutionary history, including what differentiates humans from other primates. In order to investigate how conserved or divergent miRNA are within primates, we sequenced miRNA from thirteen species, greatly expanding the number of experimentally validated non-human primate miRNA and our knowledge of their evolution.

## Results

### MiRNA discovery

**Fig 3:**
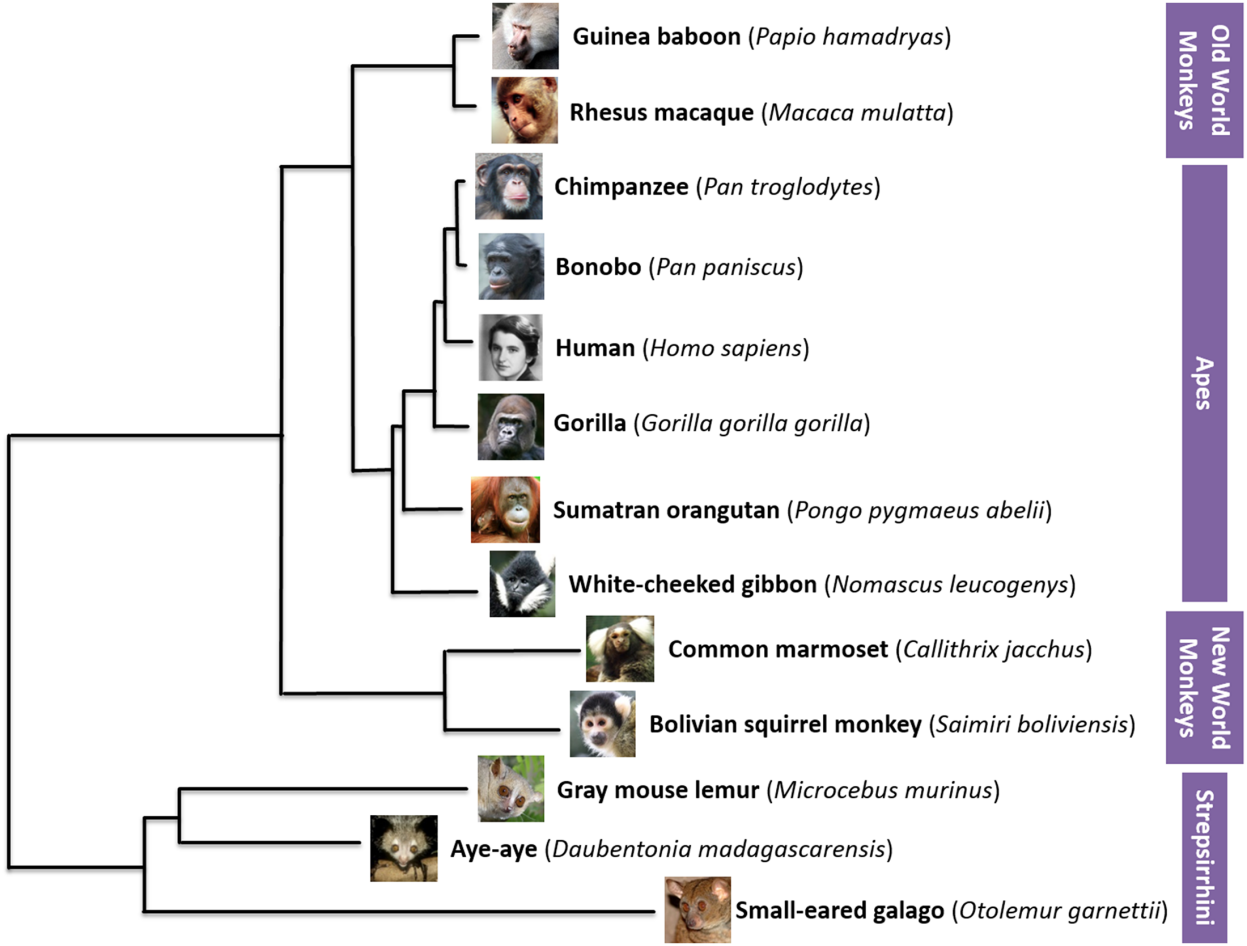
Phylogeny of primate genome assemblies included in our study (adapted from [26]). We selected species that had both a sequenced genome, and fibroblast cell culture available through Coriell Cell Repositories.

**Fig 4:**
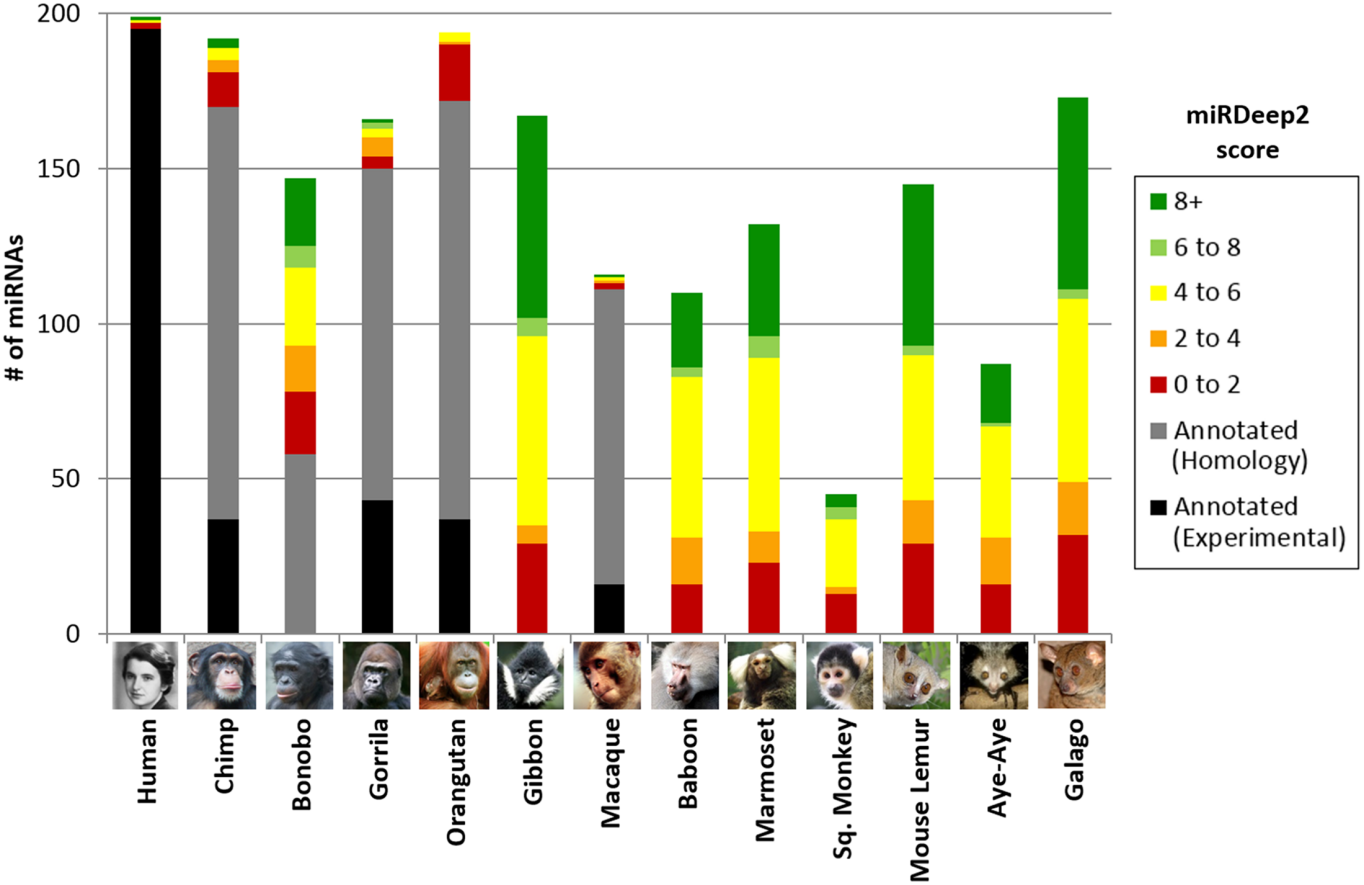
miRDeep2 results by score. MiRDeep2 scores range from -10 to 10, with a higher number corresponding to increased likelihood that the miRNA is genuine. A cut-off of 0 was used to be included in this study. MiRNA already annotated in miRBase are represented in black and gray: black represents miRNA with experimental validation, and gray represents miRNA previously predicted solely by homology to the human genome that have now been validated in this study. Novel miRNA are shown in a color corresponding to their miRDeep2 score; this score is partially determined by the availability of any previously annotated miRNA, which would inherently result in lower scores for our primates with no information in miRBase.

**Fig 5:**
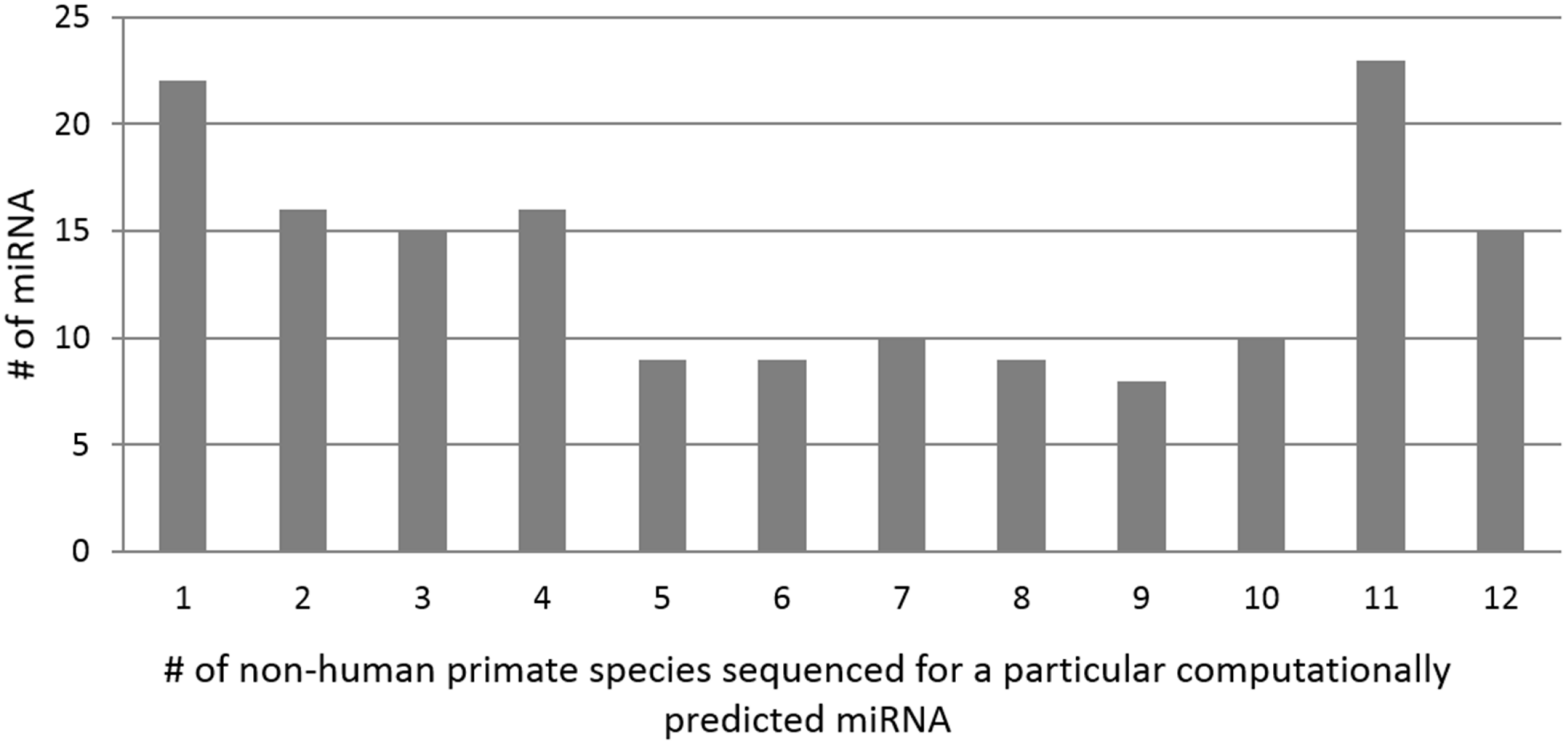
Distribution of the number of non-human primate species from our dataset sequenced for a particular miRNA that was previously computational predicted by homology alone. 140/163 (86%) have experimental support from at least two primates. Because of the difficulty distinguishing between paralogs with identical mature sequences, only the paralog with the most coverage from a family of miRNA is shown in this chart.

In order to better characterize patterns of miRNA evolution across primates, small RNAseq was performed on fibroblast cells cultured from 13 divergent primate species (Fig 3, see Methods). Our study drastically expanded our knowledge of primate miRNA, tripling the number of primate species with experimentally validated miRNA in miRBase (from 4 to 13), and adding dozens to hundreds of miRNA per species (Fig 4, S2 Table). This includes the only experimentally validated miRNA sequences available for bonobo, one of human’s closest evolutionary relatives; and for the first time, experimentally validated miRNA sequences are available for New World monkeys, lemurs, and a galago. For non-human primate miRNAs that to date were computationally predicted by homology alone, 27% (211/766) were sequenced in at least one of our primate species, with the majority of these sequences (86%) being represented by at least two species (Fig 5).

### Primate miRNA evolution

**Fig 6:**
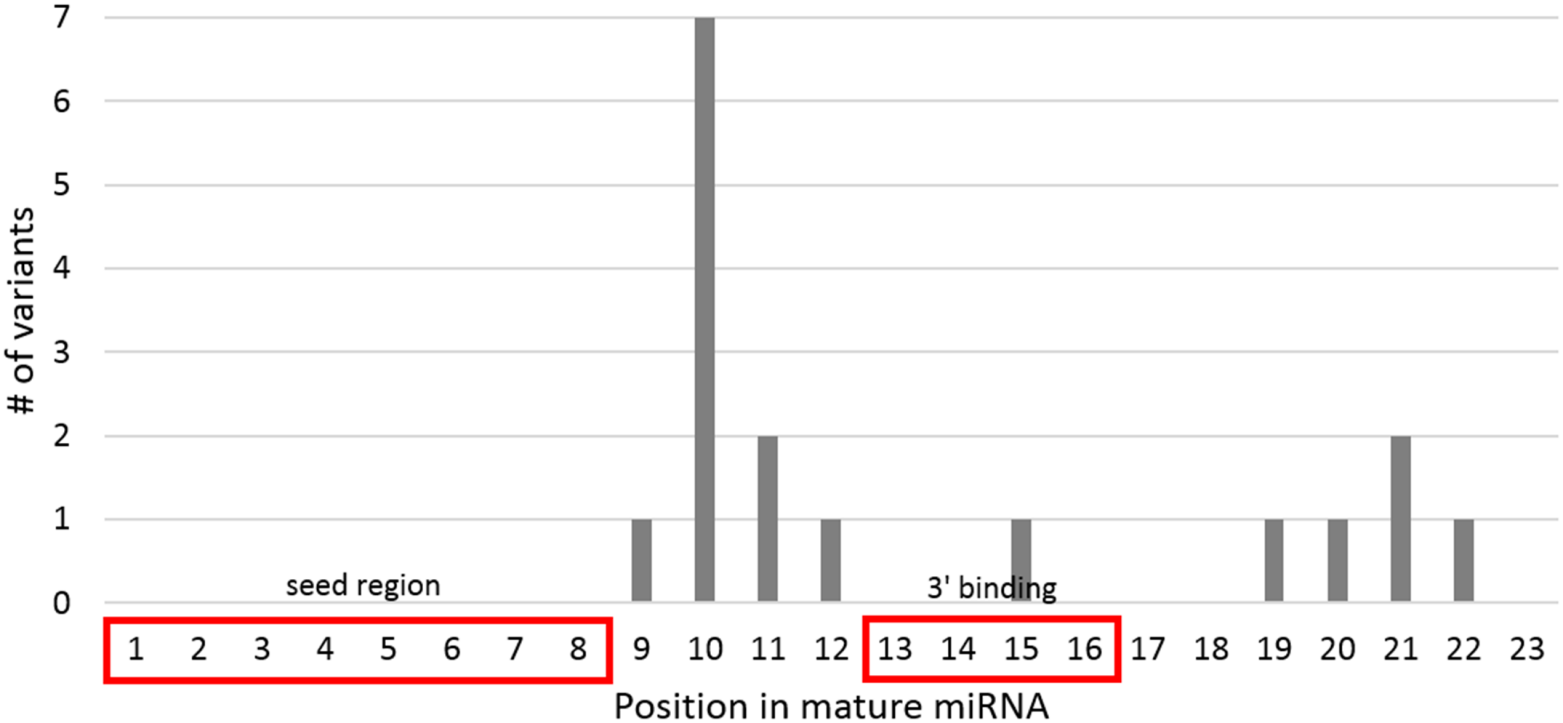
Location of variants within mature miRNA across the thirteen primate species sequenced in this study. The 5’ end of the mature miRNA has an 8 nt “seed region” in positions 1 through 8 that complementary base-pairs with the 3’ untranslated region (UTR) of messenger RNA (mRNA). The 3’ end of the mature miRNA can also have an effect: positions 13–16 are highly conserved, and their proper complementary base pairing to a mRNA target is associated with downregulation [15]. As expected, the vast majority of the variants sequenced in our study appear in positions with relaxed evolutionary constraints (positions 9–12 and 17–23).

Sequenced miRNAs were computationally clustered into groups of homologs that had at least 70% identity within the mature region (see Methods). Homology groups containing paralogs were further subdivided into their individual miRNA orthologs, resulting in 188 particular miRNA ortholog groups with representation in at least three primate species. As expected based on previous studies [19], primate miRNA appears to be highly conserved, with 173 of 188 miRNA ortholog groups (~92%) showing no variation within the mature region across primates. Of the 15 miRNA ortholog groups that contained variation within the mature region, none of these variants occurred within the seed region. This is consistent with previous studies that show the seed region to be the most highly conserved region of miRNA and the most important determinant of target recognition (Fig 6) [15]. Only one variant was found within positions 13–16, the second most conserved region of miRNA that is sometimes involved in 3’ complementary base pairing during target recognition. Most variation was observed in basal primate species: 14/21 variant sequences were from the basal Strepsirrhini suborder, and 5/21 were from New World monkeys (Table 1). This is concordant with the hominoid slowdown hypothesis, which shows that rates of nucleotide substitution in primates decrease as generation time increases [33].

**Table 1:** Summary of all variants found within the mature region of a miRNA ortholog group.

microRNA	Variant	Position	Species with variant
miR-26b-5p	T > C	11	galago
miR-501-3p	C > T	9	mouse lemur, galago
miR-28-5p	G > A	12	squirrel monkey
miR-28-5p	G > A	10	galago
miR-34b-5p	C > A	10	mouse lemur, galago
miR-193b-5p	T > A	10	mouse lemur, aye-aye, galago
miR-532-5p	C > T	20	galago
miR-151b-3p	A > G	10	mouse lemur
miR-151b-3p	G > A	11	squirrel monkey
miR-328	T > C	22	mouse lemur
miR-299-3p	C > T	10	human
miR-224-5p	G > A	19	squirrel monkey
miR-195-5p	A > T	10	galago
miR-450b-5p	A > T	10	squirrel monkey
miR-2355-5p	C > T	15	orangutan
miR-374a-5p	T > C	21	galago
miR-539-5p	T > C	21	squirrel monkey

### Structural analysis

**Fig 7:**
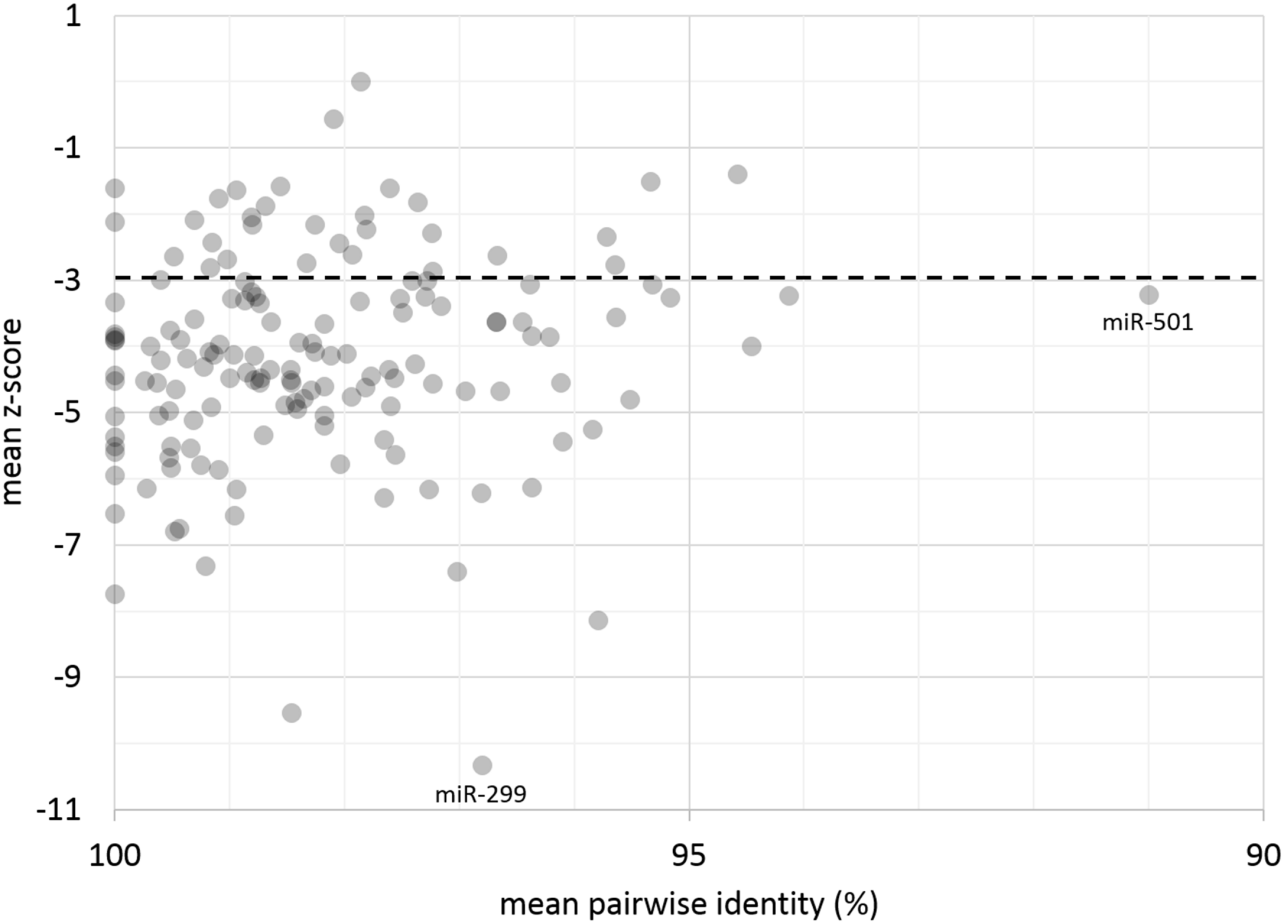
Mean pairwise sequence identity compared to the z-score, where a more negative z-score indicates increased structural stability. Scores below -3 (represented by the dotted black line) generally indicate very stable structures.

**Fig 8:**
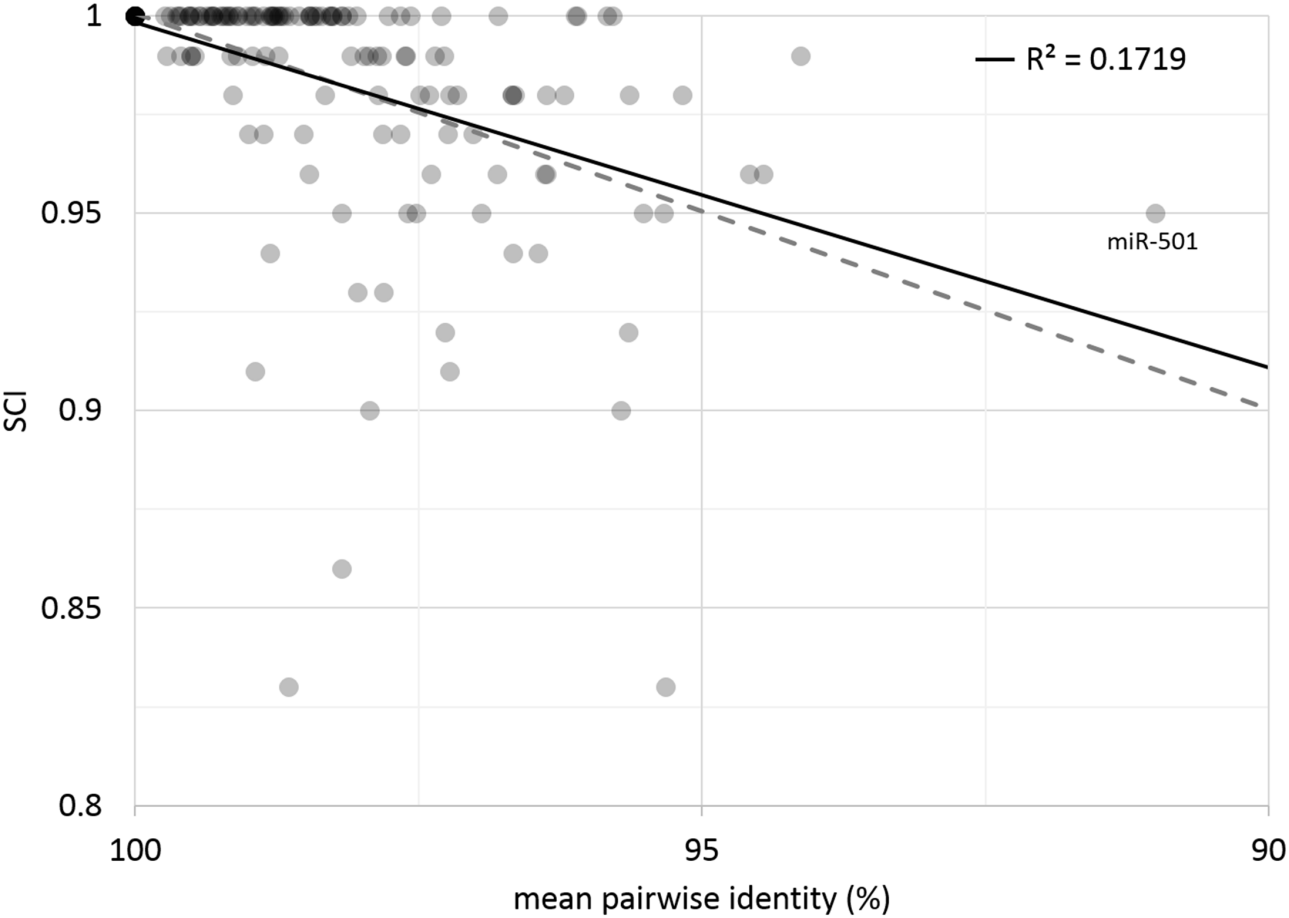
Mean pairwise sequence identity compared to the Structure Conservation Index (SCI). In general, an SCI near or above the mean pairwise identity indicates structural conservation (dotted gray line). The black line is the linear regression for our data (R^2^ = 0.1719).

We analyzed the thermodynamic stability and structural conservation of any miRNA with at least 5 species in its alignment (n = 152, see Methods). Our pre-miRNA structures are thermodynamically stable as measured by z-score (where more negative values indicate stability), with most of the analyzed miRNAs (120/152) having a z-score that indicates very conserved structures (z < -3.0) (Fig 7). Structural stability is further evidence that a miRNA sequence is genuine [34]. Structural conservation as measured by the Structural Conservation Index (SCI), where an SCI of 1 indicates complete structural conservation, generally decreases as sequence divergence increases (Fig 8), but this correlation is weak (R^2^ = .1719). This is to be expected, as SCI only approximately captures true structure conservation, but is also concordant with the properties of miRNA: a miRNA with low sequence identity may still be structurally conserved due to compensatory mutations, or a miRNA with high sequence identity may have few variants that result in drastic (and perhaps functionally significant) structural difference.

### miR 2355-5p

**Fig 9:**
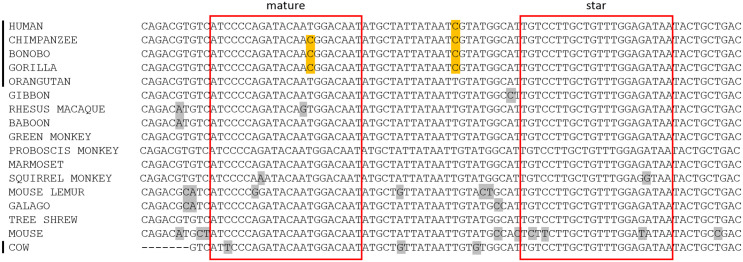
Alignment of miR-2355 homologous sequences. The black line indicates species that have experimental support for the transcription of miR-2355, either in miRBase (human, cow) or from this study (chimpanzee, bonobo, gorilla, and orangutan). The red boxes outline the mature and star sequences within the miRNA. Variants only found among the great apes are highlighted in yellow, while all other variants are marked in grey. Humans have experienced a reversion at position 15 of the mature miRNA, restoring that nucleotide to its ancestral state.

MiR-2355-5p was the only alignment to contain a variant within the conserved 3’ binding location (positions 13–16) of the mature miRNA. Homologous sequences from additional primate species and a mouse (*Mus musculus*) outgroup extracted from the UCSC Genome Browser reveal an interesting evolutionary event: the closest sister taxa to humans (chimpanzee, bonobo, and gorilla) have variant T15C in the mature sequence, while humans have seemingly reverted to the ancestral T (Fig 9) [35]. This reversion is conserved across humans in dbSNP [36]. This specific transition, as well as other mutations throughout the pre-miRNA, do not seem to alter the secondary structure of the hairpin. This is the first time RNAseq has confirmed the expression of this miRNA in a non-human primate (chimpanzee, bonobo, gorilla, and orangutan); the only other species confirmed to express this miRNA is cow (*Bos taurus*) [37], suggesting it is likely to be expressed in other primates as well. Human miR-2355-5p has been shown to be expressed in embryonic stem cells [38], neural stem cells [39], and throughout the female reproductive tract [40, 41]. The specific targets and function of miR-2355-5p are currently unknown.

### miR-299-3p

miR-299-3p was completely identical across all primates except for a single change (C10T) in humans. The secondary structure of miR-299 was entirely conserved (SCI = 1) and was the most thermodynamically stable hairpin of all the miRNA in this study (z = -10.33). Previous research identified this change based on sequences from human, chimpanzee, and macaque, and we show that the ancestral sequence is shared across all primates except human. Initial research found that miR-299-3p has human-specific expression, with preferential expression in neurons; while targets of this miRNA were enriched for neuronal function and axon guidance, there was no difference in target specificity between the human and chimpanzee versions [42]. A more recent study confirmed that miR-299-3p is expressed in cerebellum and has targets enriched for neuronal function; however, changes in target repertoire and expression levels between humans and non-human primates were identified, illustrating how a change outside of the seed region can still have profound effects [43].

### miR-501-3p

**Fig 10:**
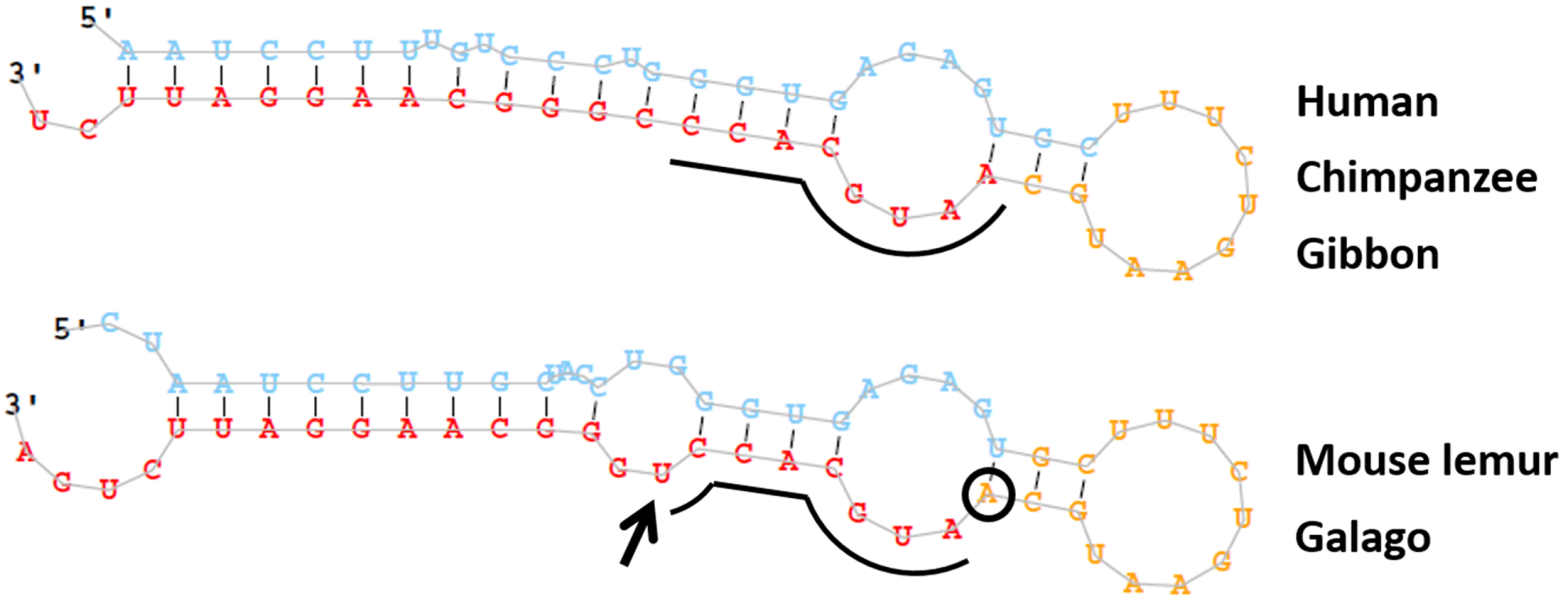
Predicted structure of miR-501 by miRDeep2. Red indicates the mature miR-501-3p sequence supported by reads, yellow the predicted loop, and blue the predicted star sequence. In mouse lemur and galago, the mature sequence contains a variant (arrow) immediately following the seed region (underlined); this as well as variants outside of the mature sequence appear to alter the overall secondary structure of the hairpin, resulting in the mature sequence and thus the seed region being shifted downstream by 1 nt (circled in black).

Of all of the alignments, miR-501 had the lowest mean pairwise identity (91%) across primates, similar to the average pairwise identity between most human and mouse pre-miRNA (>90%) [19]. However, miR-501 still appeared to be structurally conserved due to a number of compensatory mutations (SCI = 0.95, covariance contribution = -0.24). The mature sequence miR-501-3p also contains a variant just outside the seed region at position 9 in the basal Strepsirrhini suborder that introduces an additional bulge in the hairpin. This variant, as well as variants outside of the mature sequence, likely alters the overall secondary structure of the hairpin, resulting in the mature sequence being shifted downstream by 1 nt in apes relative to Strepsirrhines (Fig 10). This would alter the dicer cleavage position and shift the seed region by one nt, likely changing the target repertoire of this particular miRNA.

Previous research has shown that miR-501-3p localizes to dendrites and plays a key role in NMDA-induced dendritic spine remodeling, which is thought to be the “structural basis of information storage in the brain for cognitive functions such as learning and memory” [42]. Suppression of *GluA1* expression is necessary for long term maintenance of NMDA-induced spine modifications: experimental assays showed that miR-501-3p targets the transcript of *GluA1*, and their expression are inversely correlated during postnatal brain development. NDMA stimulation increased the expression of the primary miR-501 transcript, and still increased mature miR-501-3p levels even when a transcription inhibitor was present, suggesting that miR-501-3p undergoes post-transcriptional regulation [42]. This regulation may be controlled by the structure of miR-501, as hairpin structural stability increases the production of the mature miRNA [22]. The sampled apes in our study have increased complementary base pairing throughout the hairpin, lacking the mid-mature bulge found in Strepsirrhines. This structural difference and its resulting seed shift may indicate an important moment in primate brain evolution.

### miR-320

**Fig 11:**
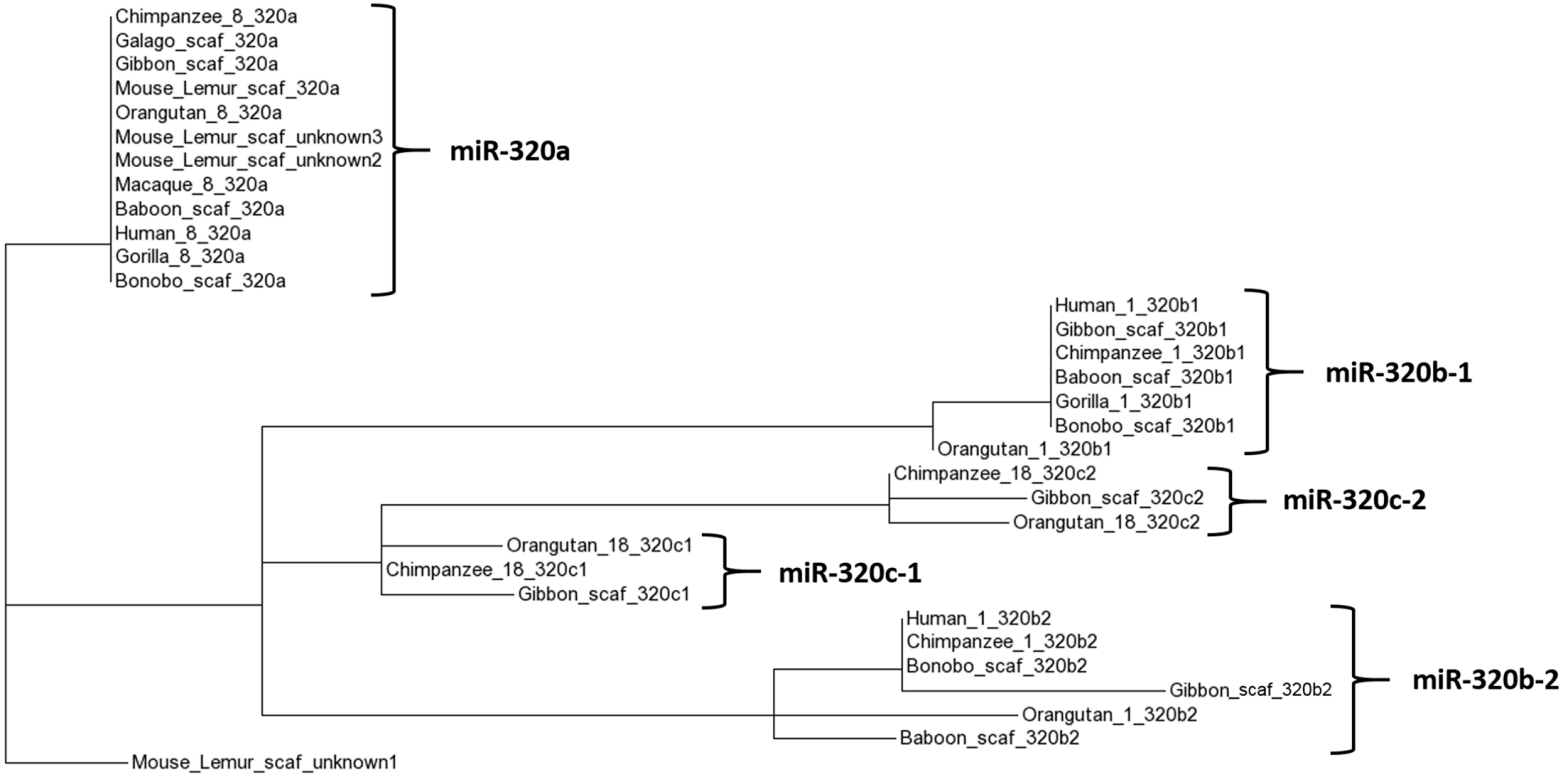
Phylogenetic tree of the predicted pre-miRNAs of the miR-320 family, based on experimentally determined mature sequences. Paralogs miR-320b and miR-320c are only expressed in apes and Old World monkeys, lacking representation from New World monkeys and Strepsirrhines.

One of our largest homology groups was composed of the miRNA-320 paralogs (Fig 11). In humans, the miR-320 family consists of one copy of miR-320a (chr8), two copies of miR-320b (chr1), two copies of miR-320c (chr18), two copies of miR-320d (chr13 & chrX), and a single copy of 320e (chr19). Gene duplication allows novel functions to evolve, as one paralog is maintained by purifying selection for its previous function while other copies are allowed to acquire mutations and neofunctionalize and/or subfunctionalize. While our data showed miR-320a to be present across the entire primate lineage, we only identified RNAseq reads for miR-320b and miR-320c in apes and Old World monkeys, matching the copy number found in humans (we did not sequence any copies of miR-320d or miR-320e from any species, likely because they are not expressed in this particular cell type). This pattern of only being found in the most derived species is unlikely to occur by random chance, and may indicate that these additional paralogs do not exist in these genomes.

**Fig 12:**
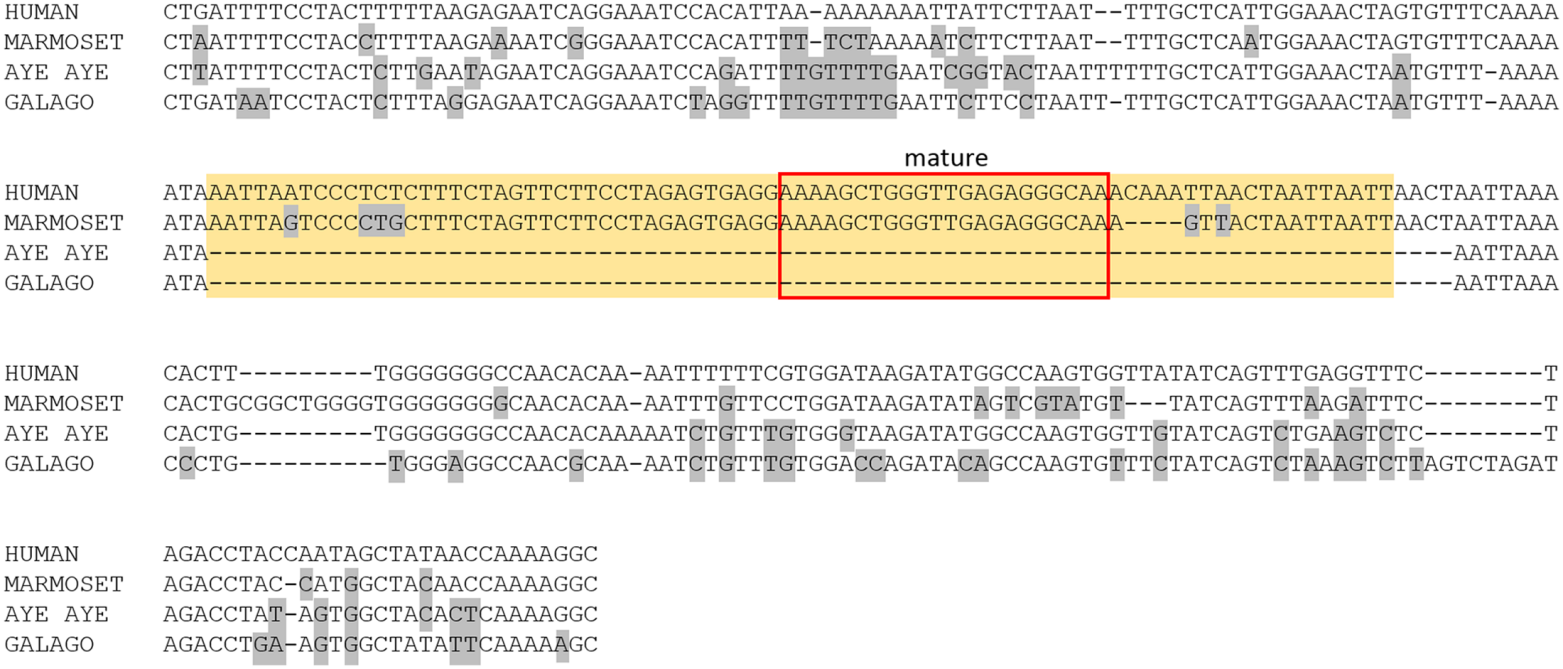
Alignment of miR-320b1 homologous sequences. The yellow box denotes the pre-miRNA sequence, red outlines the mature sequence, and variants with respect to humans are marked in grey. miR-320b1 is found in all apes, Old World monkeys, and New World monkeys (only human and marmoset are shown for simplicity). The entire pre-miRNA sequence is clearly absent in Strepsirrhines (aye-aye and galago), despite conservation of flanking sequence, demonstrating an insertion event that took place after the Strepsirrhini suborder split from the rest of the primate lineage.

miR-320a is mammalian-specific, and its paralogs have only been identified in previous studies (experimentally or computationally) in primates: 320b in gorilla, 320b and 320c in macaque, and all paralogs in chimpanzee and orangutan [27, 44, 45, 46]. We confirmed the presence or absence of each miR-320 paralog using blastn (see Methods, S3 Table). 320a was present in all primates, concordant with our RNAseq data. 320b1, 320c1, 320c2 and 320d2 were present in all primates except for the basal Stepsirrhini suborder. 320b2, 320d1, and 320e were present only in apes and Old World monkeys, with 320e having an additional duplication in orangutan. Alignments clearly indicated whether or not a sequence was present; for example, the pre-miRNA of miR-320b1 is absent in Strepsirrhines despite the conservation of flanking sequence, illustrating the insertion event that took place sometime after the Strepsirrhini suborder split from the rest of the primate lineage (Fig 12). Paralogs found in a genome but missing in our sequencing data may be identified in future RNAseq efforts; however, it is also possible that despite being in the genome, they are not expressed, are expressed only in different cell types, or are not successfully processed into mature miRNA. Our data indicates that the miR-320 family has undergone multiple gene duplications throughout primate evolution, and suggests that only more derived species successfully express mature forms of miR-320b and miR-320c, but more extensive investigation of expression in other species is needed to expand on these conclusions.

Numerous studies have illustrated the role of the miR-320 family as a regulator of neural development. The family is highly expressed in rat neurons, with enrichment specifically in axons [47], and is also targeted by REST, a transcription factor that silences neuronal genes in non-neuronal tissue and is essential in neuronal differentiation as well as the maintenance of neural stem cells [48, 49]. Transfection experiments confirm that miR-320b inhibits expression of neuron-related mRNA targets, and *in situ* hybridizations to investigate histological expression patterns show miR-320b co-localized with neurons in both human and macaques [50]. Increased levels of miR-320b have also been shown to increase neurite length, further suggesting that increased copies of this miRNA may play a role in neuronal development [51]. The miR-320 family is also frequently dysregulated in neurological disorders: miR-320 was downregulated in blood of schizophrenia patients [52] and the striatum of forebrains of Huntington Disease patients [53], and upregulated in the cortex of patients with sporadic Alzheimer's disease [54] and mouse brains undergoing prion-induced neurodegeneration [55]. In both control and Huntington Disease forebrains, miR-320b and 320c have such high rates of post-transcriptional nucleotide substitutions compared to their primary transcripts that researchers have suggested these edited sequences be considered the reference miRNA in brain tissue; additionally, these two paralogs were the only miRNA with substitutions at several positions across the mature sequence [53]. In addition to its role in neuronal development, miR-320c appears to have a wide range of functions, including regulation of chondrocytes in cartilage [56], differentiation of skeletal stem cells [57], inhibition of proliferation, migration, and invasion in bladder cancer [58], and induction of resistance to the chemotherapeutic agent gemcitabine in pancreatic cancer [59]. Given the number of duplications this miRNA family has undergone throughout primate evolution and the enrichment of neuronal functions among its paralogs, it is likely that the miR-320 family plays a role in primate brain evolution.

## Discussion

In recent decades, our understanding of functional genomics has been facilitated by technological advances in high throughput transcriptomic and proteomic methods. These tools are also rapidly expanding our understanding of miRNA, a relatively new class of trans-acting regulators of gene expression that are quickly being recognized as potential sources of phenotypic variation or as biomarkers for disease. Reliable sequence information across a diverse range of species is required for researchers to comprehensively study miRNA evolution.

In this study, we have greatly expanded the number of experimentally validated non-human primate miRNAs, especially in more divergent sister taxa. Inclusion of New World monkeys, lemurs, and a galago in this study made it possible to identify more ancient evolutionary events that have shaped primate evolution, such as the mature sequence shift we identified in miR-501-3p and the duplications of the miR-320 family. We have also demonstrated that more than a fourth of all computationally predicted primate miRNAs were found within our data (despite having only sequenced one cell type), lending confidence to prediction by homology as a method of miRNA discovery. However, our results also illustrate the importance of validating mature miRNA through RNAseq: mature miRNA sequence shifts caused by changes in secondary structure cannot be reliably determined by homology alone, and require sequencing reads to determine their boundaries.

Because this study only sequenced miRNA from a single cell type (cultured fibroblasts), we likely captured a subset of the miRNAs expressed within each species. However, it is notable that fibroblasts and neurons are both derived from the ectoderm [60], and conversion of fibroblasts to neurons is relatively easy [61]; additionally, fibroblasts are frequently used as a model when studying brain disorders because of their neuron-like signal transduction pathways [62, 63]. This relationship between fibroblasts and neurons possibly explains the abundance of neuronally-expressed miRNA identified in this study. Nonetheless, even a single cell type was sufficient for finding a number of interesting evolutionary events. More sequencing efforts across a diverse range of cell types and stages of development are likely to reveal additional insights into primate evolution, especially in cell types already known to have undergone significant phenotypic changes (i.e., neuronal tissue). More research is needed to confirm the actual biological targets of these miRNA of interest, as target prediction software is not accurate given the complicated binding interactions of miRNA: TargetScan and miranda, two of the best predictive software currently available, both have false positive rates of ~25% [64]. Even target identification is starting to benefit from high throughput technology, such as expression profiling after miRNA knockdown or overexpression [65], as well as UV cross-linking miRNA-mRNA duplexes to RISC to be pulled out via immunoprecipitation [18]. We hope that this study serves as a foundation for future research into the evolution of miRNA and gene regulation in primates.

## Methods

### Primate samples

In order to represent a broad span of the primate phylogeny, we selected thirteen primate species that had sequenced genomes and fibroblast cell cultures available through Coriell Cell Repositories (Fig 3, S4 Table):

- **Apes:** human (*Homo sapiens*), chimpanzee (*Pan troglodytes*), bonobo (*Pan paniscus*), western lowland gorilla (*Gorilla gorilla gorilla*), Sumatran orangutan (*Pongo pygmaeus abelii*), northern white-cheeked gibbon (*Nomascus leucogenys*)
- **Old World monkeys:** Indian rhesus macaque (*Macaca mulatta*), Hamadryas baboon (*Papio hamadryas*)
- **New World monkeys:** Common marmoset (*Callithrix jacchus*), Bolivian squirrel monkey (*Saimiri boliviensis*)
- **Strepsirrhines:** Gray mouse lemur (*Microcebus murinus*), Aye-aye (*Daubentonia madagascarensis*), Small-eared galago (*Otolemur garnetti*)

Marmoset is the only species where the genome of a closely related species (*C*. *jacchus*) was used as the reference for the cell line available (*C*. *geoffroyi*).

### RNA sequencing

Mature miRNA from fibroblast cells was extracted using the Qiagen miRNeasy Mini Kit following the manufacturer’s protocols (Catalog #217004, Qiagen, Valencia, CA). We prepared and barcoded samples using Illumina’s TruSeq Small RNA Library Preparation Kit (Catalog #RS-200-0012, Illumina). Barcoded samples were multiplexed for 25bp paired-end sequencing on a single lane of an Illumina MiSeq. The Picard tool ExtractIlluminaBarcodes separated raw Illumina reads by barcode, and IlluminaBasecallsToFastq output the results in fastq format. Fastqc was run on the original fastq, with FastqTOSam importing the fastqs into bam format. Bam files were processed with Picard MarkIlluminaAdapters tool. The adapter-masked fastqs were put into PEAR with no minimum overlap size in order to merge the reads. Fastqc was run on merged, trimmed fastq and compared to the original fastqc to confirm that adapters were trimmed.

### MiRNA identification

miRDeep2 was used to predict novel and identify previously annotated miRNA [66]. Merged, trimmed reads were mapped to their respective genomes using the miRDeep2 mapper.pl module with the following parameters: -c -j -l 18 -m -p -s -t–v. MiRDeep2 was executed with default parameters. When making novel miRNA predictions, miRDeep2’s algorithm accounts for already known miRNAs of the species being analyzed and of any related species. We retrieved a list of known miRNAs from miRBase (release 21) for any of our primates that were in the database (*H*. *sapiens*, *P*. *troglodytes*, *P*. *paniscus*, *G*. *gorilla*, *P*. *pygmaeus*, *M*. *mulatta*), and used all known metazoan miRNAs as our “related species” reference. MiRDeep2 assigned a score from -10 to 10 to each miRNA, with a higher number corresponding to increased likelihood that the putative miRNA is functional. This score is partially determined by the availability of any known miRNA, which would inherently result in lower scores for our primates with no information in miRBase. Because of this, we chose a relaxed score cut-off of 0 and minimum read depth of 3 to include a miRNA in our analyses, with the expectation that false positives would be removed during paralog clustering and alignment.

### Confirmation of predicted miRNAs

We compiled a list of 776 different miRNAs from miRBase that lacked experimental validation in non-human primates prior to this study. Blastn (NCBI blast 2.2.21) was then used to find a conservative match (100% identity over at least 18 nt) of these sequences within our experimentally validated miRNA. If a match was found in at least one of our primate species, the miRBase name and genome coordinates were extracted from the header of the match using a custom perl script, and that particular miRNA was counted as now having experimental validation in a non-human primate. When determining whether a given primate genome contained a particular predicted miRNA, paralogs were collapsed into a single group and the highest score was taken, as it is difficult to distinguish between paralogs that have identical or nearly identical mature sequences.

### Homolog clustering

To determine which miRNA in our data set were homologous (either as orthologs or paralogs), our mature miRNA sequences were clustered by an all-versus-all search using blastn (NCBI blast 2.2.21) at a permissive e-value (1E-02). Additional e-value cut-offs were evaluated, but given the short nature of the search queries (~22 nt mature miRNAs), it was assessed that a more permissive value was necessary to find correct matches for sequences of this length. The results were filtered for matches between sequences that had 70% identity over at least 18 nt. The sequences that remained were then clustered into groups by a custom Python script, with a sequence being added to a group if it shared at least 70% identity to any other sequence in that group. In this way, every sequence found in the search was placed into a group or was identified as not having known homologs within our dataset.

### Phylogenetic analysis

The 100 largest homology groups were selected for phylogenetic analysis, with two trees generated per group: one based on our experimentally validated mature sequence, and another based on the excised sequences as predicted by miRDeep2. These excised sequences contain the actual pre-miRNA plus ~20 nt of flanking sequence on either side. Specifically, miRDeep2 searches for the highest local stack of mature reads and excises it twice, once with 20 nt upstream and 70 nt downstream flanking sequence, and once with 70 nt upstream and 20 nt downstream flanking sequence. This is in order to determine if the mature reads occur on the 5’ or 3’ part of the hairpin, with miRDeep2 attempting to fold both excised sequences into stable hairpins. Thus, any excised sequence that is confirmed to include a pre-miRNA will have exactly 20 nt flanking on one side, and ~20 nt flanking the other (exact length of this flank can vary depending on the length of the mature, star, and loop sequences). This flanking sequence adds robustness to our alignments of already very short sequences. Sequences were aligned using the Fast Statistical Alignment Algorithm (FSA) [67]. Trees were then generated using RAxML with the following parameters: -f a -m GTRGAMMA -p 12345 -x 12345 -# 1000 -s -n [68]. Mature and excised miRNA alignments were visualized with the Max Plank Institute’s Bionformatics Toolkit [69] and trees were visualized with Phylodendron (http://iubio.bio.indiana.edu/treeapp/). Trees were visually examined for evidence of potential adaptation, such as an excess of paralogs found only in a particular subgroup of primates, or basal primates whose sequences represent an intermediate step between paralogs.

These large paralog trees were then subdivided into groups that contained only one particular miRNA (in at least three species), based on visual assessment of both mature and excised miRNA alignments and trees. In nearly all cases, miRNAs were clustered into obvious ortholog groups. In rare cases where it was difficult to determine where a particular sequence belonged, miRNA sequences in the trees were searched within the miRBase database for the closest match. These searches clearly labeled known paralogs, confirming that our self-blast clustering worked as intended. After subdivision, each particular miRNA was realigned using FSA and trees reconstructed with RAxML. Each alignment was then searched for sequence variants within the mature region of a particular miRNA, as these changes are likely to have phenotypic consequences.

### Structural analysis

The exact sequence of the pre-miRNA hairpin was retrieved from the excised sequences based on the folding predictions of miRDeep2. Pre-miRNA alignments were analyzed by the Vienna RNA Package’s RNAz program [70], which predicts the secondary structure of noncoding RNA and calculates different measures of structural conservation. Thermodynamic stability of a particular secondary structure is indicated by the z-score, which is the number of standard deviations between the minimum free energy (MFE) of a sequence compared to the MFE of random sequences of the same length and base composition; RNAz circumvents this computationally intensive step by using support vector regression to estimate mean MFE and standard deviation [71]. Lower z-scores imply greater thermodynamic stability, and scores below -3 generally indicate very stable structures that are unlikely to arise by random chance. The Structure Conservation Index (SCI) is the most accurate measure of structural conservation currently available [72]. SCI compares the average minimum free energy (MFE) of individual sequences in an alignment to a consensus MFE of that alignment. This consensus MFE is weighted by a “covariance contribution,” which gives a bonus to compensatory and consistent mutations that conserve structure, and a penalty to inconsistent mutations; a negative covariance contribution indicates more compensatory mutations. A SCI close to 1 indicates structural conservation, but SCIs cannot necessarily be compared since they depend on the number of sequences in an alignment and its mean pairwise identity. In general, SCIs near or above the mean pairwise identity of the alignment indicate good candidates for conservation [73].

### Paralog confirmation

Genomic coordinates of the human pre-miRNA for each member of the miR-320 family were obtained from miRBase, and were then used to extract pre-miRNA sequences plus 1000 nt of flanking sequence on either side from the UCSC Genome Browser. These ~2080 nt sequences were then searched against our thirteen primate genomes using blastn at an e-value of 1E-10, and were permissively filtered for matches with a minimum of 70% identity over at least 300nt. Repetitive elements in the flanking regions that were found in thousands of locations within an individual genome were removed. For each species, the match with the highest blast score that overlapped with the pre-miRNA was identified as the paralog. Some species had no overlapping matches, but did have unique matches in the flanking regions; for these, the sequence containing the hypothetical location of the pre-miRNA (100 nt upstream and 200 nt downstream from the pre-miRNA start) was extracted with a custom perl script, aligned with FSA [67], and visualized with the Max Plank Institute’s Bionformatics Toolkit [69] in order to investigate conservation of the pre-miRNA.

## Data access

miRBase policy requires acceptance of publication prior to submission. Accession numbers will appear here once processed by miRBase.

## Supporting information

S1 Table Current primate submissions in miRBase release 21 (June 2014). (XLSX)

S2 Table Number of raw reads and miRNA identified. (XLSX)

S3 Table Blastn results for miR-320 paralogs. (XLSX)

S4 Table Coriell cell repository catalog IDs. (XLSX)

## References

[1] George RD, McVicker G, Diederich R, Ng SB, MacKenzie AP, Swanson WJ, et al. 2011. Trans genomic capture and sequencing of primate exomes reveals new targets of positive selection. *Genome Res* 21: 1686–1694. doi: 10.1101/gr.121327.111 21795384 PMC3202285

[2] The Chimpanzee Sequencing and Analysis Consortium. 2004. Initial sequence of the chimpanzee genome and comparison with the human genome. *Nature* 437: 69–87. 10.1038/nature04072 16136131

[3] Goode DL, Cooper GM, Schmutz J, Dickson M, Gonzales E, Tsai M, et al. 2010. Evolutionary constraint facilitates interpretation of genetic variation in resequenced human genomes. *Genome Res* 20: 301–310. doi: 10.1101/gr.102210.109 20067941 PMC2840986

[4] King MC and Wilson AC. 1975. Evolution at two levels in humans and chimpanzees. *Science* 188: 107–116. 1090005 10.1126/science.1090005

[5] Enard W, Khaitovich P, Klose J, Zöllner S, Heissig F, Giavalisco P, et al. 2002. Intra- and interspecific variation in primate gene expression patterns. *Science* 296: 340–343 doi: 10.1126/science.1068996 11951044

[6] Lee C, Risom T, Strauss WM. 2007. Evolutionary conservation of microRNA regulatory circuits: An examination of microRNA gene complexity and conserved microRNA-target interactions through metazoan phylogeny. *DNA Cell Biol* 26: 209–218. doi: 10.1089/dna.2006.0545 17465887

[7] Pai AA, Bell JT, Marioni JC, Pritchard JK, Gilad Y. 2011. A genome-wide study of DNA methylation patterns and gene expression levels in multiple human and chimpanzee tissues. *PLoS Genet* 7: e1001316. doi: 10.1371/journal.pgen.1001316 21383968 PMC3044686

[8] Yang S, Yalamanchili HK, Li X, Yao KM, Sham PC, Zhang MQ, et al. 2011. Correlated evolution of transcription factors and their binding sites. *Bioinformatics* 27: 2972–2978. doi: 10.1093/bioinformatics/btr503 21896508

[9] Chen K and Rajewsky N. 2007. The evolution of gene regulation by transcription factors and microRNAs. *Nat Rev Genet* 8: 93–103. doi: 10.1038/nrg1990 17230196

[10] He L and Hannon GJ. 2004. MicroRNAs: small RNAs with a big role in gene regulation. *Nat Rev Genet* 5: 522–531. doi: 10.1038/nrg1379 15211354

[11] Berezikov E. 2011. Evolution of microRNA diversity and regulation in animals. *Nat Rev Gen* 12: 846–860. 10.1038/nrg3079 22094948

[12] Li Y and Kowdley KV. 2012. MicroRNAs in common human diseases. *Genomics Proteomics Bioinformatics* 10: 246–253. doi: 10.1016/j.gpb.2012.07.005 23200134 PMC3611977

[13] Fang Z, Du R, Edwards A, Flemington EK, Zhang K. 2013. The sequence structures of human microRNA molecules and their implications. *PLOS One* 8: e54215. doi: 10.1371/journal.pone.0054215 23349828 PMC3548844

[14] Nilsen TW. 2007. Mechanisms of microRNA-mediated gene regulation in animal cells. *Trends Genet* 23: 243–249 doi: 10.1016/j.tig.2007.02.011 17368621

[15] Grimson A, Farh KK, Johnston WK, Garrett-Engele P, Lim LP, Bartel DP. 2007. MicroRNA targeting specificity in mammals: determinants beyond seed pairing. *Mol Cell*. 27: 91–105. doi: 10.1016/j.molcel.2007.06.017 17612493 PMC3800283

[16] Friedman RC, Farh KK, Burge CB, Bartel DP. 2009. Most mammalian mRNAs are conserved targets of microRNAs. *Genome Res* 19: 92–105. doi: 10.1101/gr.082701.108 18955434 PMC2612969

[17] Conant GC and Wolfe KH. 2008. Turning a hobby into a job: how duplicated genes find new functions. *Nat Rev Genet* 9: 938–950. doi: 10.1038/nrg2482 19015656

[18] Hausser J and Zavolan M. 2014. Identification and consequences of miRNA–target interactions—beyond repression of gene expression. *Nat Rev Genet* 15: 599–612. doi: 10.1038/nrg3765 25022902

[19] Pang KC, Frith MC, Mattick JS. 2006. Rapid evolution of noncoding RNAs: lack of conservation does not mean lack of function. *Trends Genet* 22: 1–5. doi: 10.1016/j.tig.2005.10.003 16290135

[20] Altuvia Y, Landgraf P, Lithwick G, Elefant N, Pfeffer S, Aravin A, et al. 2005. Clustering and conservation patterns of human microRNAs. *Nucleic Acids Res* 33: 2697–2706. doi: 10.1093/nar/gki567 15891114 PMC1110742

[21] Berezikov E, Guryev V, van de Belt J, Wienholds E, Plasterk RH, Cuppen E. 2005. Phylogenetic shadowing and computation identification of human microRNA genes. *Cell* 120: 21–24. doi: 10.1016/j.cell.2004.12.031 15652478

[22] Gong J, Tong Y, Zhang HM, Wang K, Hu T, Shan G, et al. 2012. Genome-wide identification of SNPs in microRNA genes and the SNP effects on microRNA target binding and biogenesis. *Hum Mutat* 33: 254–263. doi: 10.1002/humu.21641 22045659

[23] Duan R, Pak C, Jin P. 2007. Single nucleotide polymorphism associated with mature miR-125a alters the processing of pri-miRNA. *Hum Mol Genet* 16: 1124–1131. doi: 10.1093/hmg/ddm062 17400653

[24] Han J, Lee Y, Yeom KH, Nam JW, Heo I, Rhee JK, et al. 2006. Molecular basis for the recognition of primary microRNAs by the Drosha-DGCR8 complex. *Cell*. 125: 887–901. doi: 10.1016/j.cell.2006.03.043 16751099

[25] Sun G, Yan J, Noltner K, Feng J, Li H, Sarkis DA, et al. 2009. SNPs in human miRNA genes affect biogenesis and function. *RNA*. 15: 1640–1651. doi: 10.1261/rna.1560209 19617315 PMC2743066

[26] Perelman P, Johnson WE, Roos C, Seuánez HN, Horvath JE, Moreira MA, et al. 2011. A molecular phylogeny of living primates. *PLoS Genet* 7: e1001342. doi: 10.1371/journal.pgen.1001342 21436896 PMC3060065

[27] Kozomara A and Griffiths-Jones S. 2011. miRBase: integrating microRNA annotation and deep-sequencing data. *Nucleic Acids Res* 39: 152–157. 10.1093/nar/gkq1027 PMC3013655 21037258

[28] Pritchard CC, Cheng HH, Tewari M. 2012. MicroRNA profiling: approaches and considerations. *Nat Rev Genet* 13: 358–369. doi: 10.1038/nrg3198 22510765 PMC4517822

[29] Gkirtzou K, Tsamardinos I, Tsakalides P, Poirazi P. 2010. MatureBayes: A probabilistic algorithm for identifying the mature miRNA within novel precursors. *PLoS One* 5: e11843. doi: 10.1371/journal.pone.0011843 20700506 PMC2917354

[30] Berezikov E, Thuemmler F, van Laake LW, Kondova I, Bontrop R, Cuppen E, et al. 2006. Diversity of microRNAs in human and chimpanzee brain. *Nature Genetics*. 38: 1375–1377. doi: 10.1038/ng1914 17072315

[31] Hu HY, Guo S, Xi J, Yan Z, Fu N, Zhang X, Menzel C, et al. 2011. MicroRNA expression and regulation in human, chimpanzee, and macaque brains. *PLoS Genet* 7: e1002327. doi: 10.1371/journal.pgen.1002327 22022286 PMC3192836

[32] Zhang R, Peng Y, Wang W, Su B. 2007. Rapid evolution of an X-linked microRNA cluster in primates. *Genome Res* 17: 612–617. doi: 10.1101/gr.6146507 17416744 PMC1855169

[33] Li WH and Tanimura M. 1987. The molecular clock runs more slowly in man than in apes and monkeys. *Nature* 326: 93–9. doi: 10.1038/326093a0 3102974

[34] Bonnet E, Wuyts J, Rouzé P, Van de Peer Y. 2004. Evidence that microRNA precursors, unlike other non-coding RNAs, have lower folding free energies than random sequences. *Bioinformatics* 20: 2911–2917. doi: 10.1093/bioinformatics/bth374 15217813

[35] Speir ML, Zweig AS, Rosenbloom KR, Raney BJ, Paten B, Nejad P, et al. 2016. The UCSC Genome Browser database: 2016 update. *Nucleic Acids Res* 44: D717–D725. doi: 10.1093/nar/gkv1275 26590259 PMC4702902

[36] Sherry ST, Ward MH, Kholodov M, Baker J, Phan L, Smigielski EM, et al. 2001. dbSNP: the NCBI database of genetic variation. *Nucleic Acids Res* 29: 308–311. 11125122 10.1093/nar/29.1.308 PMC29783

[37] Glazov EA, Kongsuwan K, Assavalapsakul W, Horwood PF, Mitter N, Mahony TJ. 2009. Repertoire of bovine miRNA and miRNA-like small regulatory RNAs expressed upon viral infection. *PLoS One* 4: e6349. doi: 10.1371/journal.pone.0006349 19633723 PMC2713767

[38] Hansen TB, Bramsen JB, Kjems J. 2010. Re-inspection of small RNA sequence datasets reveal several novel human miRNA genes. *PLoS One* 5: e10961. doi: 10.1371/journal.pone.0010961 20532037 PMC2881037

[39] Goff LA, Davila J, Swerdel MR, Moore JC, Cohen RI, Wu H, et al. 2009. Ago2 immunoprecipitation identifies predicted microRNAs in human embryonic stem cells and neural precursors. *PLoS One* 4: e7192. doi: 10.1371/journal.pone.0007192 19784364 PMC2745660

[40] Witten D, Tibshirani R, Gu SG, Fire A, Lui WO. 2010. Ultra-high throughput sequencing-based small RNA discovery and discrete statistical biomarker analysis in a collection of cervical tumours and matched controls. *BMC Biol* 8: 58. doi: 10.1186/1741-7007-8-58 20459774 PMC2880020

[41] Creighton CJ, Benham AL, Zhu H, Khan MF, Reid JG, Nagaraja AK, et al. 2010. Discovery of novel microRNAs in female reproductive tract using next generation sequencing. *PLoS One* 5: e9637. doi: 10.1371/journal.pone.0009637 20224791 PMC2835764

[42] Hu Z, Zhao J, Hu T, Luo Y, Zhu J, Li Z. 2015. miR-501-3p mediates the activity-dependent regulation of the expression of AMPA receptor subunit GluA1. *J Cell Biol* 208: 949–959. doi: 10.1083/jcb.201404092 25800054 PMC4384731

[43] Gallego A, Melé M, Balcells I, García-Ramallo E, Torruella-Loran I, Fernández-Bellon H, et al. 2016. Functional Implications of Human-Specific Changes in Great Ape microRNAs. *PLoS One* 11: e0154194. doi: 10.1371/journal.pone.0154194 27105073 PMC4841587

[44] Dannemann M, Nickel B, Lizano E, Burbano HA, Kelso J. 2012. Annotation of primate miRNAs by high throughput sequencing of small RNA libraries. BMC Genomics 13: 116. doi: 10.1186/1471-2164-13-116 22453055 PMC3328248

[45] Brameier M. 2010. Genome-wide comparative analysis of microRNAs in three non-human primates. *BMC Res Notes* 3: 64. doi: 10.1186/1756-0500-3-64 20214803 PMC2850348

[46] Baev V, Daskalova E, Minkov I. 2009. Computational identification of novel microRNA homologs in the chimpanzee genome. *Comput Biol Chem*. 33: 62–70. doi: 10.1016/j.compbiolchem.2008.07.024 18760970

[47] Natera-Naranjo O, Aschrafi A, Gioio AE, Kaplan BB. 2010. Identification and quantitative analyses of microRNAs located in the distal axons of sympathetic neurons. *RNA* 16: 1516–1529 doi: 10.1261/rna.1833310 20584895 PMC2905752

[48] Otto SJ, McCorkle SR, Hover J, Conaco C, Han JJ, Impey S, et al. 2007. A new binding motif for the transcriptional repressor REST uncovers large gene networks devoted to neuronal functions. *J Neurosci* 27: 6729–6739. doi: 10.1523/JNEUROSCI.0091-07.2007 17581960 PMC6672685

[49] Gao Z, Ding P, Hsieh J. 2012. Profiling of REST-dependent microRNAs reveals dynamic modes of expression. *Front Neurosci* 6: 67. doi: 10.3389/fnins.2012.00067 22590451 PMC3349273

[50] Somel M, Liu X, Tang L, Yan Z, Hu H, Guo S, et al. 2011. MicroRNA-driven developmental remodeling in the brain distinguishes humans from other primates. *PLoS Biol* 9: e1001214. doi: 10.1371/journal.pbio.1001214 22162950 PMC3232219

[51] White RE and Gifford RG. 2012. MicroRNA-320 induces neurite outgrowth by targeting ARPP-19. *Neuroreport* 23: 590–595. doi: 10.1097/WNR.0b013e3283540394 22617447 PMC3367035

[52] Vachev TI, Popov NT, Stoyanova VK, Ivanov HY, Minchev DS. 2016. Down regulation of MIR-320 gene family members in the peripheral blood of schizophrenia patients. *Int J Curr Microbiol App Sci* 5: 221–230. doi: 10.20546/ijcmas.2016.501.020

[53] Martí E, Pantano L, Bañez-Coronel M, Llorens F, Miñones-Moyano E, Porta S, et al. 2010. A myriad of miRNA variants in control and Huntington’s disease brain regions detected by massively parallel sequencing. *Nucleic Acids Res* 38: 7219–7235. doi: 10.1093/nar/gkq575 20591823 PMC2978354

[54] Hébert SS, Horré K, Nicolaï L, Papadopoulou AS, Mandemakers W, Silahtaroglu AN, et al. 2008. Loss of microRNA cluster miR-29a/b-1 in sporadic Alzheimer's disease correlates with increased BACE1/β-secretase expression. *Proc Natl Acad Sci USA* 105: 6415–6420. doi: 10.1073/pnas.0710263105 18434550 PMC2359789

[55] Saba R, Goodman CD, Huzarewich RL, Robertson C, Booth SA. 2008. A miRNA signature of prion induced neurodegeneration. *PLOS One* 3: e3652. doi: 10.1371/journal.pone.0003652 18987751 PMC2575400

[56] Ukai T, Sato M, Akutsu H, Umezawa A, Mochida J. 2012. MicroRNA-199a-3p, microRNA-193b, and microRNA-320c are correlated to aging and regulate human cartilage metabolism. *J Orthop Res* 30: 1915–1922. doi: 10.1002/jor.22157 22674437

[57] Hamam D, Ali D, Vishnubalaji R, Hamam R, Al-Nbaheen M, Chen L, et al. 2014. microRNA-320/RUNX2 axis regulates adipocytic differentiation of human mesenchymal (skeletal) stem cells. *Cell Death Dis* 5: e1499. doi: 10.1038/cddis.2014.462 25356868 PMC4237271

[58] Wang X, Wu J, Lin Y, Zhu Y, Xu X, Xu X, et al. 2014. MicroRNA-320c inhibits tumorous behaviors of bladder cancer by targeting Cyclin-dependent kinase 6. *J Exp Clin Cancer* Res 33:69. doi: 10.1186/s13046-014-0069-6 25178497 PMC4431489

[59] Iwagami Y, Eguchi H, Nagano H, Akita H, Hama N, Wada H, et al. 2013. miR-320c regulates gemcitabine-resistance in pancreatic cancer via SMARCC1. *Br J Cancer* 109: 502–511. doi: 10.1038/bjc.2013.320 23799850 PMC3721395

[60] Chang C and Hemmati-Brivanlou A. 1998. Cell fate determination in embryonic ectoderm. J *Neurobiol* 36: 128–151. 9712300

[61] Vierbuchen T, Ostermeier A, Pang ZP, Kokubu Y, Südhof TC, Wernig M. 2010. Direct conversion of fibroblasts to functional neurons by defined factors. *Nature* 463: 1035–1041. doi: 10.1038/nature08797 20107439 PMC2829121

[62] Manier DH, Shelton RC, Ellis TC, Peterson CS, Eiring A, Sulser F. 2000. Human fibroblasts as a relevant model to study signal transduction in affective disorders. *J Affect Disord* 65: 51–58. 10.1016/s0165-0327(99)00190-1 11099740

[63] Garbett KA, Vereczkei A, Kálmán S, Brown JA, Taylor WD, Faludi G, et al. 2015. Coordinated messenger RNA/microRNA changes in fibroblasts of patients with major depression. *Biol Psychiatry* 77: 256–265. doi: 10.1016/j.biopsych.2014.05.015 25016317 PMC4254393

[64] Mazière P and Enright AJ. 2007. Prediction of microRNA targets. *Drug Discov Today* 12: 452–458. doi: 10.1016/j.drudis.2007.04.002 17532529

[65] Thomas M, Lieberman J, Lal A. 2010. Desperately seeking microRNA targets. *Nat Struct Mol Biol* 17: 1169–1174. doi: 10.1038/nsmb.1921 20924405

[66] Friedländer MR, Chen W, Adamidi C, Maaskola J, Einspanier R, Knespel S, et al. 2008. Discovering microRNAs from deep sequencing data using miRDeep. *Nat Biotechnol* 26: 407–415. doi: 10.1038/nbt1394 18392026

[67] Bradley RK, Roberts A, Smoot M, Juvekar S, Do J, Dewey C, et al. 2009. Fast statistical alignment. *PLoS Comput Biol* 5:e1000392. doi: 10.1371/journal.pcbi.1000392 19478997 PMC2684580

[68] Stamatakis A. 2014. RAxML Version 8: a tool for phylogenetic analysis and post-analysis of large phylogenies. *Bioinformatics* 30: 1312–1313. doi: 10.1093/bioinformatics/btu033 24451623 PMC3998144

[69] Biegert A, Mayer C, Remmert M, Söding J, Lupas A. 2006. The MPI Bioinformatics Toolkit for protein sequence analysis. *Nucleic Acids Res* 34: W335–339. doi: 10.1093/nar/gkl217 16845021 PMC1538786

[70] Lorenz R, Bernhart SH, Höner Zu Siederdissen C, Tafer H, Flamm C, Stadler PF, et al. 2011. ViennaRNA Package 2.0. *Algorithms Mol Biol*. 6: 26. doi: 10.1186/1748-7188-6-26 22115189 PMC3319429

[71] Gruber AR, Findeiß S, Washietl S, Hofacker IL, Stadler PF. 2009. RNAz 2.0: Improved noncoding RNA detection. *Pac Symp Biocomput* 69–79. doi: 10.1142/9789814295291_0009 19908359

[72] Gruber AR, Bernhart SH, Hofacker IL, Washietl S. 2008. Strategies for measuring evolutionary conservation of RNA secondary structures. *BMC Bioinformatics*. 9: 122. doi: 10.1186/1471-2105-9-122 18302738 PMC2335298

[73] Washietl S. 2011. RNAz 2.1 user manual. https://www.tbi.univie.ac.at/~wash/RNAz/manual.pdf

